# Protective Effects of Four Structurally Distinct Sanshools Ameliorate Dextran Sodium Sulfate-Induced Ulcerative Colitis by Restoring Intestinal Barrier Function and Modulating the Gut Microbiota

**DOI:** 10.3390/antiox13020153

**Published:** 2024-01-25

**Authors:** Zhaojun Chen, Hui Wang, Lulin Tan, Xiong Liu

**Affiliations:** 1 College of Food Science, Southwest University, Chongqing 400715, China; chenzhaojun8811@163.com; 2 Guizhou Provincial Academy of Agricultural Sciences, Guiyang 550000, Chinatanlulin01@163.com (L.T.)

**Keywords:** sanshool, colitis, intestinal barrier, p65, intestinal flora

## Abstract

Hydroxy-α-sanshool (HAS), hydroxy-β-sanshool (HBS), hydroxy-γ-sanshool (HRS), and γ-sanshool (RS) are the key components from the *Zanthoxylum* genus, processing a range of pharmacological activities. The present study investigated the protective capacities of four sanshools on a dextran sulfate sodium (DSS)-induced model of ulcerative colitis (UC). The results showed that sanshool administration alleviated the colitis symptoms by reducing body weight loss and disease activity index (DAI) score, increasing the colon length, and improving colonic injury and the change in immune organ weight. Furthermore, sanshools enhanced the antioxidant enzyme activities, and RS exhibited the lowest effect on the improvement in total antioxidative capacity (T-AOC) and antioxidant abilities compared to the other three sanshools. The p65 nuclear factor κB (p65 NFκB) signaling pathway was inhibited to prevent hyperactivation and decreased the production of inflammatory factors. The gut barrier function in DSS-induced mice was restored by increasing goblet cell number and levels of tight junction proteins (zonula occludens-1, occludin, and claudin-1), and the levels of protein in HAS and HRS groups were higher than that in the HBS group, significantly. The analysis of gut microbiota suggested that sanshool administration significantly boosted the abundance of *Lachnospiraceae*, *Muribaculaceae*, *Oscillospiraceae*, and *Alistipes* and reduced the level of *Buchnera* in colitis mice. Collectively, the sanshool treatment could ameliorate colitis by resisting colon injury and regulating intestinal barrier dysfunction and gut microbiota dysbiosis; meanwhile, HRS and HAS have better improvement effects.

## 1. Introduction

Ulcerative colitis (UC) is a subtype of chronic inflammatory bowel disease (IBD) with the ulcer formation of mucosa in the colon and rectum [1]. The pathogenesis involves an interaction between environmental, genetic, and immunological factors [2]. The incidence of UC in Western developed countries was much higher than that in developing countries, which were 19.2 and 24.3/100,000 in Canada and Northern Europe, respectively [3]. However, it has risen rapidly in the East over the recent years. The symptoms of UC are severe diarrhea, frequent rectal, and blood in the stool, accompanied by different complications in the lung, liver, mucosa, skin, and other parts, bringing huge stress and economic burden to patients [4]. The treatment of UC uses corticosteroids, thiopurines, 5-aminosalicylic acid, and molecular targeted agents, depending on the disease severity. However, these conventional therapies are accompanied by toxicity and side effects, hormone dependence, and recurrence after drug withdrawal, causing serious problems for patients and clinicians [5].

Multiple studies have demonstrated that there is a close relationship between intestinal microbiota and UC pathogenesis. Significant reductions in the relative abundance of the *Firmicutes* have been observed in UC patients. The *Firmicutes* seem to be the important producers of short-chain fatty acid metabolites (acetate and butyrate) with potent anti-inflammatory properties [6]. In addition, the disruption of microbiota caused a rapid increase in harmful bacteria, such as *Coprococcus*, *Peptostreptococcus*, and *Eubacteria,* suggesting that these members could play collective or individual roles in disease pathology, although they still need to be certified [7]. The growing harmful bacteria directly damage and invade intestinal epithelial cells, resulting in damage to metabolic and energy metabolism, triggering intestinal mucosa injury and inflammation. These microbial pathogens are *Mycobacterium avium paratuberculosis*, *Clostridium* (*C.*) *dofficile*, *Helicobacter* species, adherent-invasive *Escherichia coli*, *Salmonella* species, *Fusobacterium* species, norovirus, *Yersinia* species, and *Listeria* species [8], indicating that intestinal microbiota is essential for the occurrence of UC. The use of medicinal plants or herbs in UC is increasing worldwide, developing as an alternative or complementary therapy for many patients. The aloe vera gel, topical Xilei-san, Andrographis paniculate extract, and Triticum aestivum were adept in remission clinical response; Boswellia serrata gum resin was as effective as mesalazine [9]; Artemisia absinthium, mastic gum, and Tripterygium wilfordii were excellent in preventing clinical postoperative recurrence [8].

Sanshools are long-chained polyunsaturated amides produced from the *Zanthoxylum* genus and characterized by three conjugated double bonds at the end of the fatty acid chain. A growing collection of biological effects of the *Zanthoxylum* genus were attributed to sanshools, including anticancer, antibacterial, anti-inflammatory, antioxidant, and hepatoprotective [10]. The *Zanthoxylum* alkyl amides hydroxy-α-sanshool and hydroxy-β-sanshool as the important components were responsible for the induction of colonic motor activity, contraction of smooth muscle cells, and enhancement of defecation rates [11]. Several studies showed that the Zanthoxylum fruit extract was particularly associated with antibacterial activity against the tested strains, such as *Escherichia coli*, *Staphylococcus coli*, *Bacillus laterosporus Laubach*, *Bacillus subtilis*, and *Bacillus cereus.* The minimum bactericidal and the minimum inhibitory concentration were 2.5–20 mg/mL and 1.25–5.0 mg/mL, respectively [12]. However, the effect of sanshools on UC-improving through intestinal microbiota regulation has never been studied.

In traditional medicines, *Zanthoxylum* has been used as an anti-inflammatory agent, but the detailed clinical mechanism of sanshools is severely lacking. In the present study, the key components, *Zanthoxylum* genus, HAS, HBS, HRS, and RS, were chosen to perform a detailed evaluation of protective effects on intestinal tracts of UC mice, according to structural difference contrast among four sanshools. We measured relevant factors, including body weight change, disease activity index, inflammation cytokines, antioxidant enzyme activity, and levels of protein involved in intestinal barrier function. The underlying mechanism was measured by high-throughput sequencing of gut microflora to provide better project choices for the treatment of UC.

## 2. Materials and Methods

### 2.1. Chemicals and Reagents

Four sanshools (purity ≥ 98%) were obtained from Medson Technology Co., Ltd. (Chengdu, China). Dextran sodium sulfate (DSS, molecular weight 36,000–50,000 kDa) was provided by Aladdin Biochemical Technology Co., Ltd. (Shanghai, China). Superoxide dismutase (SOD), catalase (CAT), glutathione peroxidase (GSH-Px), T-AOC, malondialdehyde (MDA) kits, and ELISA kits of interleukin-6 (IL-6), tumor necrosis factor-α (TNF-α), interleukin-10 (IL-10), and interleukin-1β (IL-1β) were all obtained from Jiancheng Bioengineering Institute (Nanjing, China). Primary antibodies (anti-ZO-1, anti-occludin, anti-claudin-1, anti-p65, anti-p-p65 anti-IκB, anti-p-IκB, anti-actin) and secondary antibodies were obtained from the Biyotime Institute of Biotechnology (Shanghai, China).

### 2.2. Animals and Experimental Design

**Figure 1:**
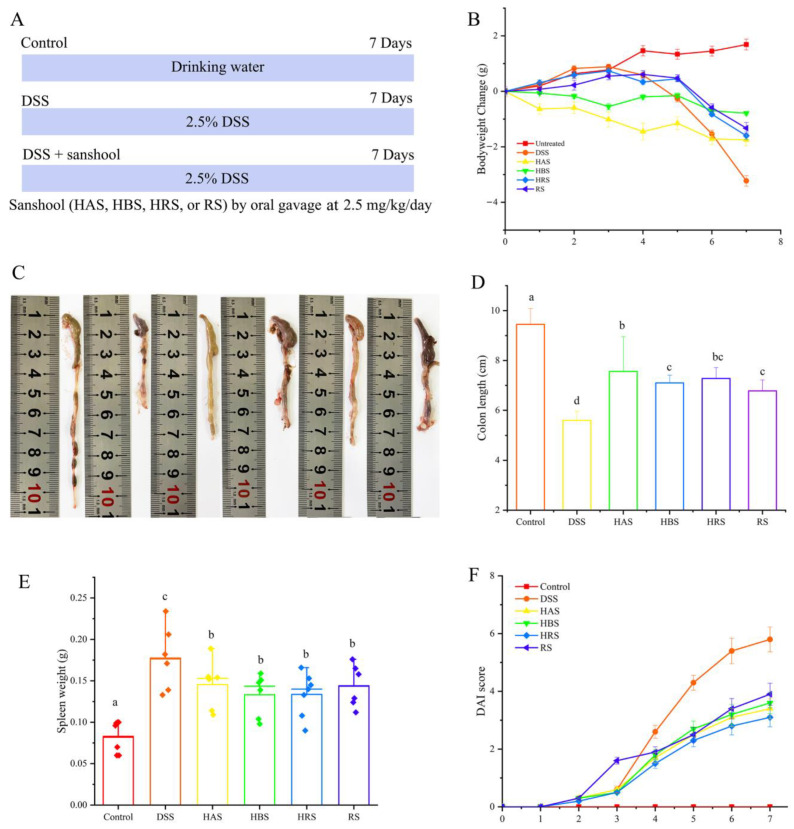
Effect of sanshools on the pathological symptoms of UC mice. (**A**) Schematic diagram of sanshools supplementation; (**B**) Body weight changes of the mice; (**C**,**D**) Colon length; (**E**) Weight of spleen; (**F**) Disease activity index (DAI) score. The results are shown as mean ± SD (*n* = 6–8 per group), and the values without the same letter represent significantly different at *p* < 0.05.

Animal studies were conducted according to protocols approved by the Guizhou University Subcommittee of Experimental Animal Ethics on 8 December 2022. The ethical committee’s identification number was EAE-GZU-2022-E025. Forty-eight specific pathogen-free (SPF) male C57BL/6J mice (20 ± 2 g) were acquired from Spife Biotechnology Co., Ltd. (Beijing, China), given free access to food and water at a temperature of 23 ± 2 °C and kept for 12 h dark/light cycle. After acclimatization period, the mice were randomly divided into 6 groups (*n* = 8), containing normal control group (Control), DSS-induced UC model group (DSS), DSS + hydroxy-α-sanshool group (HAS), DSS + hydroxy-β-sanshool group (HBS), DSS + hydroxy-γ-sanshool group (HRS), and DSS + γ-sanshool group (RS). All mice except the Control group were offered drinking water with 3% (*w*/*v*) DSS, and the gavage doses of four sanshools were all at 2.5 mg/kg/d. The sanshool addition (<3 mg/kg/d) was safe and nontoxic in the mice models, according to our previous study [13,14]. All mice were weighed and observed daily, and euthanasia by excessive isoflurane was on the eighth day. After photographing and measuring the colon length, part of the colon was fixed in 4% paraformaldehyde, and the remaining colon was immediately frozen at −80 °C for future studies. The mouse spleen tissues were weighed. The experimental protocol is presented in Figure 1A.

### 2.3. Evaluation of Disease Activity Index (DAI)

DAI was measured according to the study of Zhang et al. [15] and obtained by calculating the body weight loss, bloody stools, and diarrhea. The scoring criteria were shown as follows: body weight loss (0, none; 1, 0–5%; 2, 5–10%; 3, 10–15%; 4, >15%); bloody stools (0, normal; 2, hemoccult positive; 4, severe bleeding); diarrhea (0, normal; 2, loose stool; 4, diarrhea). The DAI was the average of three indicators score.

### 2.4. Histological Assessments and Immunofluorescence Staining

Distal colonic tissues were washed with ice NS, embedded by paraffin, and sectioned into 5 μM thicknesses. After being dewaxed, these sections were subjected to hematoxylin and eosin (H&E) and Alcian blue staining. The slides were observed by an inverted optical microscope (Nikon 80i, Tokyo, Japan). The images were used for pathological analysis, and the evaluation rules were based on the study of Du et al. [16]. The number of goblet cells was obtained by Image J Version 1.0 software (Bethesda, MD, USA). Immunofluorescence staining of tight junction proteins was performed according to the previous study [15].

### 2.5. Serum and Colonic Parameter Measurements

The levels of serums T-AOC, SOD, MDA, and GSH-Px, as well as colon CAT, were carried out using biochemical kits. The serum levels of TNF-α, IL-1β, IL-10, and IL-6 were measured using ELISA kits. All parameters were determined according to the manufacturers’ instructions.

### 2.6. Western Blot Assessments

The colon tissues were homogenized after adding the radioimmunoprecipitation assay (RIPA) buffer with 1 mM phenylmethylsulfonyl fluoride (PMSF) obtained from the Beyotime Institute of Biotechnology (Shanghai, China). The supernatant of lysates was gathered after 13,000× *g* centrifugation at 4 °C for 15 min, and the protein concentration was measured with a BCA assay kit (Solarbio, Beijing, China). The collected protein samples were separated with a 10% SDS polyacrylamide gel and transferred to polyvinylidene difluoride membranes. Next, these membranes were blocked with 5% dried skim milk for 2 h and incubated overnight at 4 °C using the primary antibodies. The membranes were treated with a secondary antibody for 2 h and observed with a high-sensitivity Chemiluminescence imaging system (Bio-Rad, Hercules, CA, USA).

### 2.7. Gut Microbiota Analysis

The colonic contents were sent to Biomarker Biotechnology Co., Ltd. (Beijing, China). Total genomic DNA was extracted using a TGuide S96 Magnetic Soil/Stool DNA Kit (Tiangen Biotech (Beijing) Co., Ltd., Beijing, China). The bacterial 16S rRNA (V3-V4 region) was amplified with primers (338F: 5’-ACTCCTACGGGAGGCAGCA-3’; 806R: 5’-GGACTACHVGGGTWTCTAAT-3’). After filtering and trimming the sequencing data, the PCR products were collected, and the paired ends (2 × 250 bp) were performed on the Illumina Novaseq 6000 (Predicine, Hayward, CA, USA). The obtained data were analyzed on the BMKCloud online platform. The microbial compositions between groups were analyzed with linear discriminant analysis (LDA) effect size (LEfSe), and the filter value of LDA was set to 3.5. Species with significant differences (*p* < 0.05) were determined through SPSS version 17.0 software to conduct a between-group *T*-test and plotted.

### 2.8. Statistical Analysis

Experimental data were displayed as the mean ± standard deviation. The results were analyzed using one-way ANOVA with Duncan’s multiple range and least significant difference tests using SPSS 17 software and considered significant differences at *p* < 0.05.

## 3. Results

### 3.1. Sanshools Alleviated the Symptoms of DSS-Induced Colitis Mice

To explore the effect of sanshool on alleviating colitis, the body weight changes, DAI, colon length, and spleen weight of each mouse were observed. The body weight change of the mice in the Control group exhibited a gradual upward trend throughout the experimental period, and that of the DSS-treated mice group induced a significant decrease from the fourth day (Figure 1B). However, mice in the four sanshools groups had significantly lower reductions in body weight than mice in the DSS group on the seventh day. The colon length in the DSS group was significantly shorter than that in other treated groups. As seen in Figure 1C, the administration of HBS and RS can reduce colitis symptoms, but abnormal morphology and a lot of blood were still observed, suggesting that the improvement effects were lower than those of HAS and HRS. HAS (7.56 ± 1.4), HBS (7.1 ± 0.31), HRS (7.28 ± 0.44), and RS (6.78 ± 0.44) had 35.00%, 26.79%, 30.00%, and 21.07% larger colon lengths, respectively, than that of the DSS group (5.61 ± 0.37) (Figure 1C,D). Compared with the Control group, DSS-fed led to abnormal immune organ weight, such as an elevation in spleen weight, indicating that the inflammatory response had emerged, while gavage with sanshools obviously improved this variation (Figure 1E). In addition, as for the DAI score, it exhibited a similar variation trend. The DAI scores of all groups increased rapidly through the seven days due to the presence of DSS, except the Control group. From the fourth day, the scores for sanshool groups (HAS, HBS, HRS, and RS) were significantly lower than the DSS-induced group (Figure 1F). Altogether, the above results suggested that sanshool supplementation can effectively mitigate the pathological symptoms of colitis.

### 3.2. Sanshools Attenuate the Colonic Injury of DSS-Induced Mice

**Figure 2:**
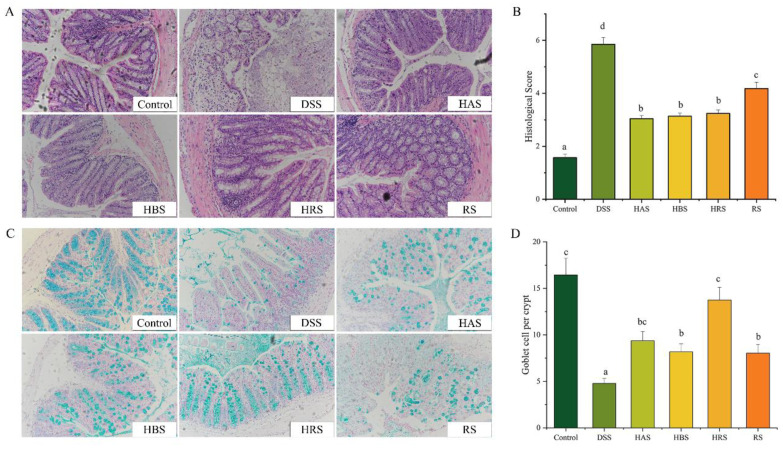
Effect of sanshools administration on colonic pathological injury of DSS-induced mice. (**A**) Representative images of H&E staining (red) in colonic tissues. (**B**) Histopathological score. (**C**) Representative images of alcian blue staining in colonic tissues (blue). (**D**) The goblet cells number of colonic tissues. The results are shown as mean ± SD (*n* = 6–8 per group), the values without the same letter represent significantly different at *p* < 0.05, and the scale bar is 200 μM.

Next, the effects of sanshools on the colonic tissue injury of DSS-induced mice were evaluated through H&E and alcian blue staining. As shown in Figure 2A, compared to the Control group, DSS-induced UC in mice caused serious colon injury, containing extensive inflammatory cell infiltration, unclear colonic stratification, crypt shortening, and incomplete structural characteristics. Therefore, the histopathological scores of DSS-induced mice were significantly higher than those of normal mice (Figure 2B). However, treatment with sanshools (HAS, HBS, HRS, and RS) diminished aberrant colonic histopathological symptoms, while the score of mice with intragastric administration of RS was higher than the other three sanshool groups. Furthermore, the results of alcian blue staining on colonic tissues displayed an obvious decrease (70.94%) in goblet cells of mice with DSS administration, indicating that DSS destroyed the production of intestinal mucosal mucus (Figure 2C). The sanshools (HAS, HBS, HRS, and RS) administration increased the number of goblet cells (by 1.96-, 1.71-, 2.88-, and 1.68-fold, respectively) in colon tissues, and the HRS group had the highest value in comparison to the other three sanshool groups (Figure 2D). These data indicated that sanshool administration can improve colonic tissue injury in DSS-induced mice.

### 3.3. Effects of Sanshools on Antioxidant Activity in DSS-Induced Colitis Mice

**Figure 3:**
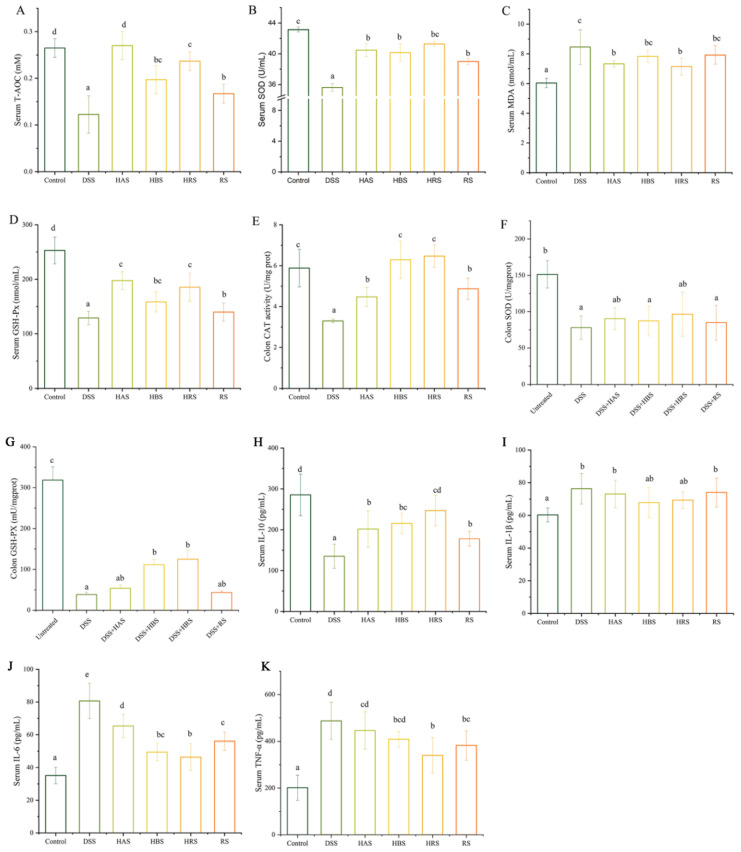
Effects of sanshools on antioxidant capacity and inflammatory cytokines in DSS-induced mice. (**A**) Serum T-AOC. (**B**) Serum SOD. (**C**) Serum MDA. (**D**) Serum GSH-Px. (**E**) Colon CAT. (**F**) Colon SOD. (**G**) Colon GSH-Px. The colon tissue was milled, lysed, centrifuged, and assayed with corresponding kits. (**H**) Serum IL-10. (**I**) Serum IL-1β. (**J**) Serum IL-6. (**K**) Serum TNF-α. The results are shown as mean ± SD (*n* = 6–8 per group), and the values without the same letter represent significantly different at *p* < 0.05.

The levels of serum T-AOC, serum SOD, serum MDA, serum GSH-Px, colon CAT, colon SOD, and colon GSH-Px were exhibited in Figure 3A–G. Compared to the Control group, the values of serum T-AOC, serum SOD, serum GSH-Px, and colon CAT in DSS-induce mice were decreased by 53.85%, 17.45%, 48.99%, and 43.88%, respectively, whereas, the value of serum MDA in DSS group was increased by 39.9%. Importantly, the levels of serum T-AOC, serum SOD, serum GSH-Px, and colon CAT in the HAS, HBS, HRS, and RS groups were significantly higher than that of the DSS group, whereas the serum MDA values of HAS and HRS were remarkably decreased. The levels of MDA in the HBS and RS groups were reduced, but there were no significant in comparison to the DSS group. In addition, the treatments with HRS and HBS increased the colon GSH-Px levels significantly. Upward trends were observed in the colon SOD of four sanshool groups, but no significance.

### 3.4. Effects of Sanshools on Inflammatory Cytokines in Serum of DSS-Induced Colitis Mice

As depicted in Figure 3H–K, the levels of TNF-α, IL-6, and IL-1β in the DSS group were significantly higher (2.42-, 2.29-, and 1.27-fold, respectively) than that in the Control group, whereas the value of IL-10 in serum was decreased significantly. Expectably, compared to the DSS group, the levels of TNF-α were decreased in the HRS and RS groups. A reduction in IL-6 levels was observed in the HAS, HBS, HRS, and RS groups, whereas IL-10 levels were increased significantly compared to the DSS group. There was a decreasing trend but no significant difference in levels of IL-1β among the DSS group and sanshool administration groups. Furthermore, in terms of the four sanshool groups, the HRS group has the minimum values of TNF-α and IL-6, as well as the maximum value of IL-10.

### 3.5. Effects of Sanshools on the Integrity of Intestinal Barrier in DSS-Induced Mice

**Figure 4:**
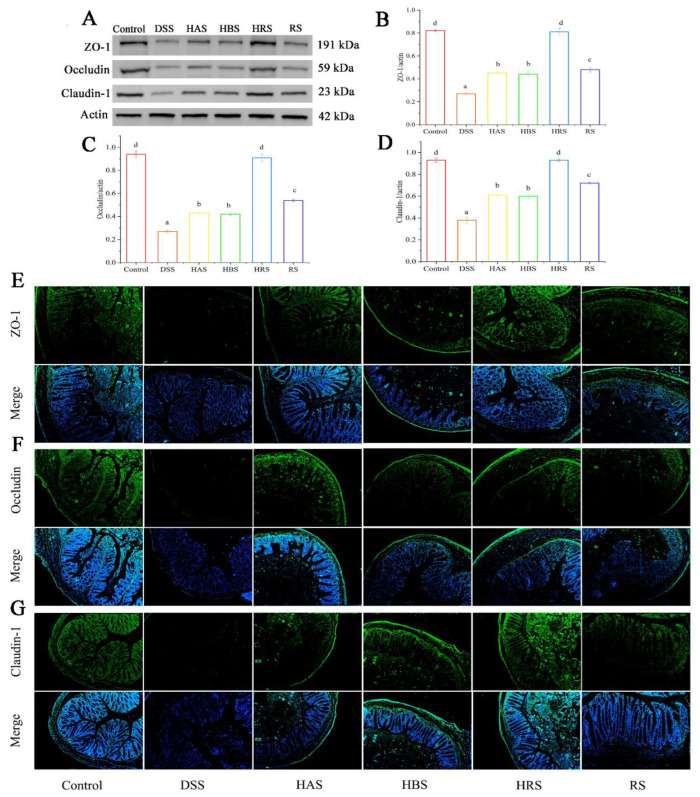
Effects of sanshools on colonic barrier integrity on DSS-induced mice. (**A**) The images of western blot of ZO-1, claudin-1, and occludin. (**B**–**D**) The gray-scale levels of TJ protein (green). (**E**–**G**) Representative immunofluorescence pictures of ZO-1, claudin-1, and occludin in colon tissues of mice. The results are shown as mean ± SD (*n* = 6–8 per group), the values without the same letter represent significantly different at *p* < 0.05, and the scale bar is 200 μM.

To research the effects of sanshools on the intestinal barrier integrity in DSS-induced mice, the protein levels of ZO-1, occludin, and claudin-1 in mouse colon tissues were carried out by analysis of western blot and immunofluorescence. The results showed that the values of ZO-1, occludin, and claudin-1 in colonic tissue of the DSS group were decreased significantly (by 75.38%, 72.60%, and 77.03%, respectively) (Figure 4A–D). However, compared to the DSS group, the tight junction (TJ) protein levels in sanshool groups (HAS, HBS, HRS, and RS) were increased remarkably: ZO-1 (by 2.69-, 2.50-, 2.81-, and 1.69-fold, respectively), occludin (by 2.45-, 2.10-, 3.6-, and 1.2-fold, respectively), and Claudin-1 (by 2.82-, 2.29-, 4.24-, and 1.76-fold, respectively). According to the above data, the HRS group exhibited the best effect of improving the integrity of the intestinal barrier in DSS-induced mice, followed by the HAS group. Similarly, the results of immunofluorescence analysis (Figure 4E–G) indicated the protective effects of sanshool administration on DSS-induced colitis by raising the expression of TJ proteins in colon tissues.

### 3.6. Effects of Sanshools on the Levels of p65 Pathway of DSS-Induced Mice

**Figure 5:**
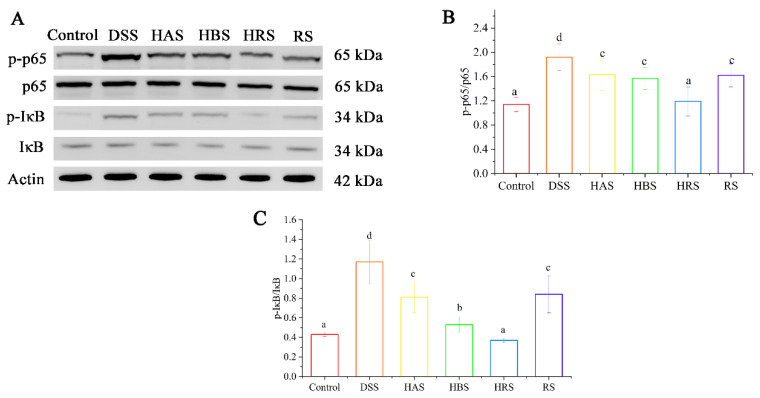
Effects of sanshools administration on p-65 signaling of DSS-induced mice. (**A**) Representative images of p65, p-p65, IκB, and p-IκB in colon tissues. (**B**,**C**) The gray-scale analysis of corresponding protein bands. The results are shown as mean ± SD (*n* = 3 per group), and the values without the same letter represent significantly different at *p* < 0.05.

The expressions of several key proteins (p65, p-p65, IκB, and p-IκB) in colonic tissues were detected to explore the mechanism underlying the improvement effect of sanshools on colonic inflammation. As compared to the Control group, the phosphorylation levels of p65 and IκB were significantly increased (2.70- and 1.34-fold, respectively) in the colonic tissues of the DSS group (Figure 5A–C). However, the administration of sanshools (HAS, HBS, HRS, and RS) decreased the levels of p-p65 (by13.70%, 67.12%, 78.08%, and 24.66%, respectively) and p-IκB (72.58%, 41.13%, 11.29%, 15.32%, respectively). In addition, the level of p-p65 in the HRS group exhibited the minimum value compared with the other three sanshool groups, and a similar result happened in the p-IκB value of the HAS group.

### 3.7. Effects of Sanshools on the Dysbiosis of Gut Microbiota in DSS-Induced Mice

**Figure 6:**
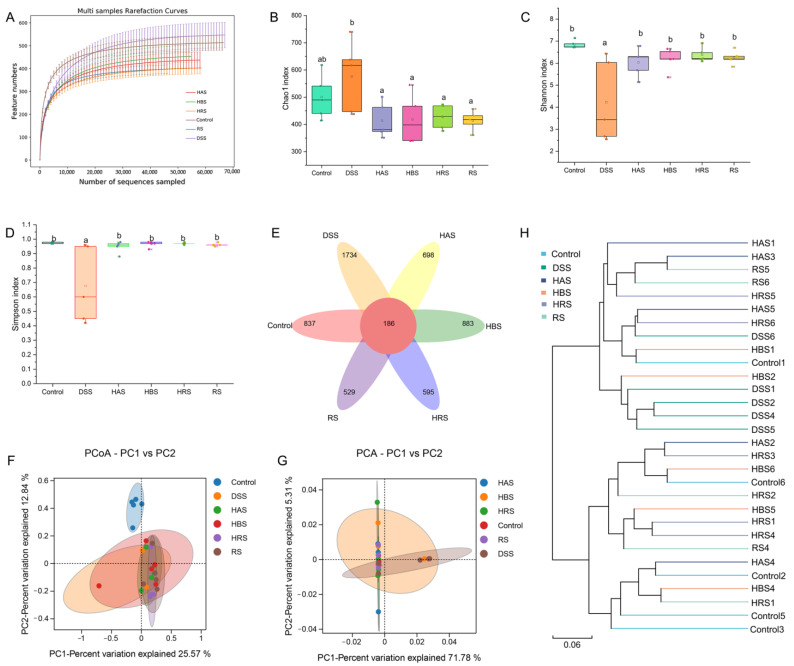
Effects of sanshools on intestinal microbiota of DSS-induced mice. (**A**) Dilution curve of intestinal flora. (**B**) Chao 1 index. (**C**) Shannon index. (**D**) Simpson index. (**E**) Venn image of bacteria at OUT level. (**F**) Result of PCoA analysis on bacterial community structure. (**G**) Result of PCA analysis on microflora. (**H**) Hierarchical clustering analysis. The results are shown as mean ± SD (*n* = 6–8 per group), and the values without the same letter represent significantly different at *p* < 0.05.

The rarefaction curve of OUT levels in the gut microbiota of mice gradually tended to be flat with the rise in the number of reads, indicating that the sample sequence amounts were reasonable enough for analysis (Figure 6A). A Venn diagram exhibited that 837, 1734, 698, 883, 595, and 529 unique OTUs were found in the Control, DSS, HAS, HBS, HRS, and RS groups, respectively (Figure 6E). Compared to the Control group, the Chao 1 index (Figure 6B) of the DSS group was increased, whereas the Shannon and Simpson indexes (Figure 6C,D) were decreased markedly. Treatment with sanshools (HAS, HBS, HRS, and RS) improved the variation in the diversity of gut microbiota in the DSS group. The beta-diversity analysis was carried out to clarify the extent of similarity between microbial communities. As shown in Figure 6F,G, an apparent clustering separation was observed between the Control and DSS groups, and the sanshool administration mitigated this DSS-induced shift. In addition, the HRS and HBS groups were closer to the Control group than the DSS group, according to the cluster analysis result (Figure 6H).

**Figure 7:**
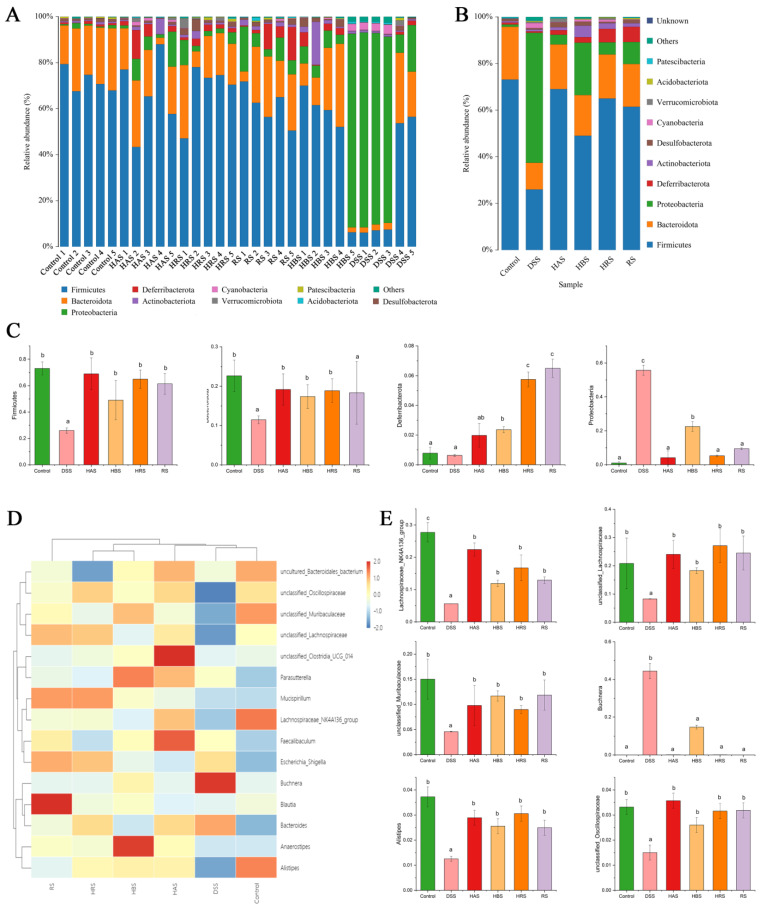
Effects of sanshools on gut microbiota composition. (**A**) Analysis of community composition of samples at phylum level. (**B**) Analysis of community composition of groups at phylum level. (**C**) Effects of sanshools on *Firmicutes, Bacteroidota, Proteobacteria,* and *Deferribacterota* at phylum level. (**D**) The heatmap of top 15 microbiota at the genus level. (**E**) Effects of sanshools on *Alistipes*, *Lachnospiraceae_NK4A136_group*, *Lachnospiraceae*, *unclassified_Muribaculaceae*, *unclassified_Oscillospiraceae*, and *Buchnera.* The results are shown as mean ± SD (*n* = 6–8 per group), and the values without the same letter represent significantly different at *p* < 0.05.

The gut microbiota composition in the fecal of different groups was further performed at variation taxonomic levels (Figure 7). At the phylum level (Figure 7A,B), the dominant bacteria were *Firmicutes, Bacteroidota, Proteobacteria*, *Deferribacterota*, *Actinobacteriota*, *Desulfobacterota*, and *Cyanobacteria*. DSS treatment decreased the relative abundance of *Firmicutes* and *Bacteroidota* and increased the levels of *Protecobacteria* compared to the Control group (Figure 7C). However, sanshools administration can reverse the variation trend of dominant bacteria in DSS-induced mice. At the genus level (Figure 7D), sanshools (HAS, HBS, HRS, and RS) administration increased the relative abundance of *Alistipes*, *Lachnospiraceae_NK4A136_group*, *unclassified_Lachnospiraceae*, *unclassified_Muribaculaceae*, and *unclassified_Oscillospiraceae* and decreased *Buchnera* abundance in comparison to the DSS group (Figure 7E).

**Figure 8:**
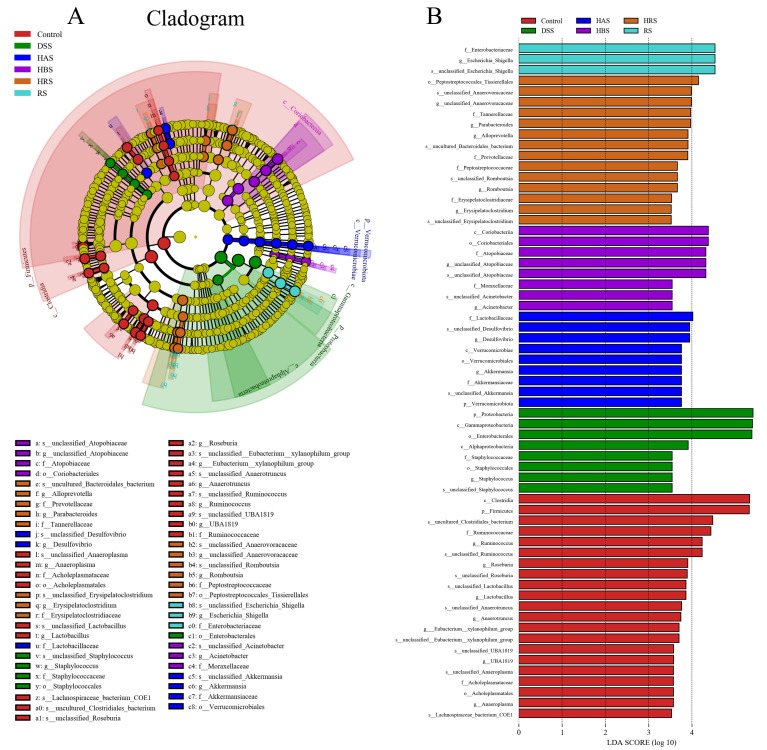
Effect of sanshools on composition of intestinal flora. (**A**) The diagram of LEfSe analysis. (**B**) LDA of LEfSe analysis (LDA > 3.5, *p* < 0.05).

Linear discriminant analysis effect size (LEfSe) can identify the differences in the compositional structure of intestinal microbiota and distinct biomarkers in different groups. As shown in Figure 8A,B, *g_Anaeroplasma*, *g_Lactobacillus*, *g_Roseburia*, and *g_Ruminococcus* were increased and used as the biomarker in the Control group, while *g_Staphylococcus* was special for the DSS group. The sanshool groups showed different results, the dominant species of the HAS group being *g_Desulfovibrio* and *g_Akkermansia*, the HBS group being *g_Acinetobacter* and *g_unclassified_Atopobiaceae*, the HRS group being *g_Parabacteroides*, *g_Alloprevotella*, *g_Romboutsia*, *and g_Erysipelatoclostridium*, and the RS being *g_Escherichia_Shigella*. These results showed that dietary sanshool consumption could regulate the community structure of gut microbiota in DSS-induced mice.

### 3.8. The Correlation between Gut Microbiota and UC-Related Parameters

**Figure 9:**
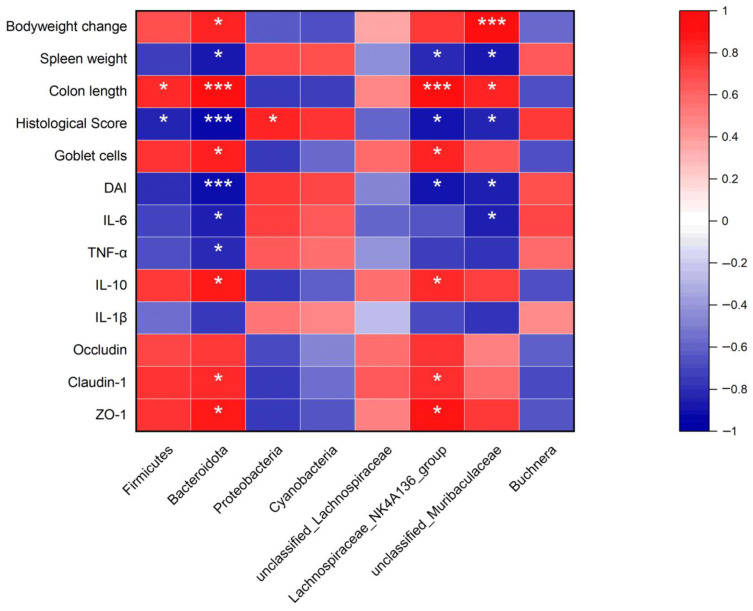
Correlation between gut microbiota and UC-related Parameters. Red color was a positive correlation, and blue color was a negative correlation. * *p* < 0.05, and *** *p* < 0.001.

The study of Spearman’s correlation was performed to illustrate the correlations between gut microbiota and key parameters in the colons of DSS-induced mice. As shown in Figure 9, at the phylum level, *Firmicutes* was positively correlated with colon length and negatively with histological score. *Bacteroidota* was negatively correlated with spleen weight, histological score, DAI, IL-6, and TNF-α and positively with bodyweight change, colon length, goblet cells, IL-10, claudin-1, and ZO-1. *Proteobacteria* was positively correlated with the histological score. At the genus level, *Lachnospiraceae_NK4A136_group* was negatively correlated with spleen weight, histological, and DAI and positively with colon length, goblet cells, IL-10, claudin-1, and ZO-1. *Muribaculaceae* was negatively correlated with spleen weight, histological score, and DAI and positively with body weight change and colon length.

## 4. Discussion

Ulcerative colitis (UC) is a chronic inflammatory bowel disorder in the colonic mucosa, characterized by a remitting and relapsing course [17]. In our present study, drinking water with 3% DSS was used to induce experimental colitis in mice for one week. On the 4th day, in the model group, it was observed the typical physiological symptoms of UC, such as weight loss, sticky stool with blood, and drooped spirits. On the day of anatomy, we observed shortened colons, larger spleens, disappeared crypt structures, and significant inflammation. These symptoms confirmed that the model of colitis mice was successfully established and consistent with previous studies [15,18,19]. A plant-based diet could prevent the occurrence and relapse of ulcerative colitis [9]. Sanshool was a natural ingredient extracted from the *Zanthoxylum* genus. This research [20] points to the primary component of *Zanthoxylum* alkamides as responsible for the benefit to intestinal health. However, the beneficial effects of dietary sanshools with protection on the DSS-induced UC mice have not yet been reported. Currently, we explored the protective effects of sanshools (HAS, HBS, HRS, and RS) from the *Zanthoxylum* genus against DSS-induced acute ulcerative colitis mice, as well as its mechanism. Our results showed that gavage with sanshools (2.5 mg/kg/d) in mice could be a potential drug for UC treatment through inhibiting inflammation, elevating the activities of antioxidants, rebuilding the gut mucosal barrier, and regulating the composition of gut microbiota. Meanwhile, the ameliorate abilities have different effects among sanshools with variations in the molecular structure.

In our study, short-term DSS induction feeding caused colitis symptoms with loss of crypts, reduction in goblet cells, depletion of the mucosal barrier, and inflammatory cell infiltration, which was similar to the previous literature [15,21]. Importantly, our research provided evidence that four sanshools with variations in structure had the ability to reverse DSS-induced colitis. It was confirmed that oxidative stress plays a vital role in the pathophysiology of colitis, and excessive oxygen radicals are important factors in inflammatory tissue injury and colitis. The mechanism is that excess reactive oxygen species (ROS) produced by oxidative stress promotes the synthesis of inflammatory cytokines by activating the pyrin domain-containing 3 (NLRP3) and nucleotide-binding oligomerization domain, causing the release of inflammasome. The inflammasome triggers p65-activated pro-inflammatory signaling transductions and further produces cytokines containing IL-6, IL-8, and IL-1β. The oxidative damage causes impairments in DNA, lipids, and protein, induced production of inflammation and aging of organisms, and decreased antioxidant enzyme activities [22]. So, the levels of antioxidant enzymes (SOD, GSH-Px, and CAT) are good indications of oxidative damage. In our study, treatment with DSS reduced the levels of antioxidant enzyme activities and T-AOC and increased the content of MDA, which were reversed by sanshool. This result was similar to previous research [23]. Furthermore, our results showed that pro-inflammation cytokines (IL-6, TNF-α, and IL-1β) were decreased and anti-inflammation cytokine (IL-10) was increased significantly, indicating sanshools have the ability to eliminate oxygen radicals and decrease intestinal oxidative damage, finally mitigating the effects of inflammation. The high levels of nuclear transcription factor kappaB (NFκB p65) were proved in epithelial cells and macrophages isolated from colon tissues of UC patients [24], and the severity of the UC symptom was remarkably correlated with the amount of activated p65 [25]. Normally, NFκB dimers are inactivated by combining with small inhibitory molecules (IκBα or IκBβ) existing in the cytoplasm [26]. Once p65 was activated through the proteasomal degradation of phosphorylation IκB in intestinal epithelial cells, it induced and controlled formation of the pro-inflammatory cytokines [27]. In our study, the levels of p-p65 and p-IκB in the DSS group were increased significantly, indicating that the p65 pathway was activated and caused UC symptoms. Therefore, this aberrant p65 and IκB phosphorylation are the targets for the treatment of chronic inflammatory diseases in the intestine. As expected, sanshools administration decreased the levels of phosphorylated p65 and IκB in colonic tissues of DSS-induced mice. Our results indicated the sanshools exhibited an excellent anti-inflammatory function through the p65 signal pathway.

The goblet cell number was reduced remarkably in the alcian blue staining of DSS group colonic tissues. It secretes resistin-like molecule-β, trefoil peptides, and mucous to defend and repair the epithelial layer, for which the main function is the integrity maintenance of the epithelial barrier [28]. The colon barrier integrity is considered to limit the entry of bacterial toxins and pathogens but allows the permeability of water, nutrients, and essential ions. However, the loss of barrier integrity will contribute to generating inflammatory symptoms in the colon [29]. Our results showed that the goblet cell number was increased significantly in the sanshool (HAS, HBS, HRS, and RS) groups, indicating that sanshool can repair the intestinal epithelial layer by increasing the goblet cell number. In addition, the barrier integrity was regulated by a tight junction (TJ), composed of several transmembrane and cytosolic proteins, containing occluding, claudin-1, and zonula occluden (ZO), and forming an architecture to perform the function of “gate and fence” [30]. The TJ proteins can tighten the space between adjacent cells, prevent the free diffusion of membrane proteins, and selectively open to molecules and solutes. The barrier defects can be induced by pro-inflammatory cytokines in two ways: first, by the regulation of protein expression and, second, by affecting the processes of redistribution [31]. In addition, the alterations in gut microbiota composition can also cause barrier disturbances. This was confirmed by different mouse models, such as IL-10 deficient mice without colitis symptoms under germ-free conditions [32]. The production of metabolites from microbiota (short-chain fatty acid) will affect the activity of a transport mechanism that changes the luminal fluid load in the colon [33]. Prior studies demonstrated that DSS induction increases intestinal permeability and reduces the expression of tight junction proteins [18,19]. Our study exhibited that TJ protein expression was also downregulated in the DSS group, similar to these studies. However, sanshools administration increased the expressions of occludin, claudin-1, and ZO-1 in DSS-induced mice.

The steady state of gut microbiota is important to prevent the occurrence and development of UC. Symbiotic bacteria of intestinal mucosal could inhibit intestinal colonization by pathogens, preventing the invasion of pathogens for the lamina propria layer [34]. Gut microbiota releases bioactive compounds containing short-chain fatty acids (acetic acid, butyric acid, and propionic acid), which act as the main energy substrates and have a strong immunomodulatory effect. When breaking the balance of intestinal microbiota, the defense and immunoregulatory function of the intestinal tract is reduced, and the relevant pathogenic factors are elevated, finally inducing intestinal mucosal invasion and aggravating colitis [8]. For these reasons, we deduced that the protective effect of dietary sanshool consumption on colitis might be related to gut microbiota. We further investigated whether sanshool consumption can regulate the gut microbiota composition of DSS-induced mice. It was found that the relative abundance of *Proteobacteria* was increased significantly in the DSS group. The studies of microbial diversity have continually confirmed an expansion of *Proteobacteria* in colitis mice and even in patients with IBD, which contains *Escherichia coli*, *enterohepatic helicobacter*, and *Campylobacter concisus*, all associated with the UC [35]. The high level of *Proteobacteria* is a microbial signature of gut dysbiosis. Importantly, sanshools treatment had inverse effects on the structure of gut microbiota and increased the relative abundance of protective bacteria (*Firmicutes* and *Bacteroidota*). In addition, the levels of *Deferribacterota* in the HBS, HRS, and RS groups were increased significantly as compared with the DSS group. Recent research [36] about energy metabolism reported that *Deferribacterota* can use carbon sources, including glycogen, maltose, sucrose, cellobiose, salicin, and arbutin. At the genus level, sanshool administration significantly boosted the abundance of *Lachnospiraceae*, *Muribaculaceae*, *Oscillospiraceae*, *Mucispirillum*, and *Alistipes*. The *Lachnospiraceae* includes known producers of butyric acid, which is a nutrient for epithelial cells, having effects of anti-inflammatory and immune modulation [37], while the *Muribaculaceae* was correlated with propionate positively [38]. *Oscillospiraceae* [39] is a producer of valeric acid, positively correlated with an anti-inflammatory response. The *Alistipes* can protect mice against colitis [40]. In addition, compared to the DSS group, the relative abundance of *Buchnera* was remarkably reduced in the sanshools group. The *buchnera* belongs to the *Proteobacteria*, which were associated with UC pathogenesis [35]. Therefore, our results showed that sanshool treatment can modulate the gut microbiota at phylum and genus levels in UC mice. In order to explain the potential role of gut microbiota in the development of colitis, the correlation between microbes and inflammation symptoms was performed. The significant microbes, such as *lachnospiraceae_NK4A136_group*, *Muribaculaceae*, and *Bacteroidota,* were prominent correlates with colitis features, gut barrier, and inflammation. The above results showed that the protective effects of sanshool administration on colitis are related to the regulation of gut microbiota.

Interestingly, RS exhibited the lowest effect on improvement of T-AOC and antioxidant enzyme activities compared to the other three sanshools. This may be due to the structure difference that hydroxy was absent in RS. The radical scavenging activity of compounds depended on the hydrogen-donating ability, which came from H-donating groups (hydroxy, sulfhydryl, and amide) [41]. According to Son et al.’ report [42], the antioxidant ability of synthetic caffeic was increased with elevating numbers of hydroxyl groups or catechol moieties in an emulsified linolenic acid oxidation system. Therefore, hydroxy is important for the regulation of antioxidant enzymes in colitis mice. For sanshool groups, the levels of TJ protein in the HRS and HAS groups were higher than in the HBS group, significantly. This may be due to the structure difference in that HRS and HAS were *cis* isomer, and HBS was *trans* isomer. According to current studies, the bioactivities, pharmacokinetics, and metabolism of *cis* and *trans* isomers of compounds were not uniform. Research of lycopene [43] clearly found human tissues contained mainly *cis* isomers, but about 90% of lycopene in dietary sources is all-*trans* conformation, and these researchers suggested *cis* isomers of lycopene were better absorbed because of the shorter length and greater solubility in mixed micelles, resulted in a lower tendency to aggregate. The study of Hubbard [44] suggested that the synthesis of rhodopsin needs *cis* isomer vitamin A and *cis* isomer retinene, both primarily all-*trans* are ineffective. However, the other report [45] showed that only all-*trans* forms of menaquinone-7 exhibited biological significance among geometric isomer forms (*cis*, *trans*, and *cis/trans*), due to *cis*-isomer with non-linear structure impairing the interaction between menaquinone-7 and subcellular structure including vitamin K2-dependent enzymes and proteins. The results showed that the ability to maintain bioactivities for *cis-trans* isomers varies in different compounds. Therefore, we provided some support for differences in the bioactivities of sanshool with various structures. 

## 5. Conclusions

In conclusion, sanshool administration has remarkable effects on attenuating intestinal mucosa inflammation, maintaining intestinal barrier homeostasis, and regulating the gut microbiota in DSS-induced mice. Therefore, our results suggest that sanshool has the potential to be an agent for the prevention and improvement of ulcerative colitis; meanwhile, HAS and HRS have better effects because of *cis* isomer and hydroxy structure.

## Data Availability

Data is contained within the article.
